# Prevalence of methicillin resistant *Staphylococcus aureus* in Lumbini Medical College and Teaching Hospital, Palpa, Western Nepal

**DOI:** 10.1186/s13104-017-2515-y

**Published:** 2017-06-02

**Authors:** Shristi Raut, Kishor Bajracharya, Janak Adhikari, Sushama Suresh Pant, Bipin Adhikari

**Affiliations:** 1 Department of Microbiology, Lumbini Medical College, Palpa, Nepal; 2 B.P. Koirala Institute of Health Sciences, Dharan, Nepal; 3 Mahidol-Oxford Tropical Medicine Research Unit, Faculty of Tropical Medicine, Mahidol University, Bangkok, Thailand

**Keywords:** MRSA, D-zone test, Cefoxitin, *Staphylococcus aureus*

## Abstract

**Background:**

Multidrug resistant *Staphylococcus aureus* is common in both tertiary and primary health care settings. Emergence of methicillin resistance in *S. aureus* (MRSA) along with macrolide, lincosamide, streptogramin B (MLSB) has made treatment of Staphylococcal infection more challenging. The main objective of this study was to detect MRSA, MLSB (inducible; MLSBi and constitutive; MLSBc) resistant *S. aureus* using phenotypic methods and to determine their antibiogram.

**Methods:**

Various samples were collected from 1981 patients who attended Lumbini Medical College and Teaching Hospital (LMCTH) during the period of 6 months from September 2015 to February 2016. Out of a total of 1981 samples, 133 *S. aureus* were isolated. Cefoxitin was used to detect MRSA by the disk diffusion test. Inducible clindamycin resistance (MLSBi) was detected by the D-zone test. The antibiotic profile of all isolates was tested by a modified Kirby Bauer disk diffusion method.

**Results:**

Among 133 *S. aureus*, there were 58 (43.6%) MRSA, 34 (25.6%) MLSBi and 30 (22.6%) MLSBc. Of a total of 64 MLSB, a significant proportion (62.5%) was MRSA (p < 0.001). Among 11 different antibiotics that were tested for *S. aureus*, MRSA showed significant resistance to 9 (p < 0.05) with the exception of vancomycin and linezolid. All the isolates were 100% sensitive to linezolid. MLSBi organisms were 100% sensitive to vancomycin and linezolid. Both MLSBi and MLSBc showed a higher degree of resistance to multiple antibiotics (p < 0.05).

**Conclusions:**

Isolation of MRSA, MLSBi and MLSBc were remarkably high. Routine use of simple and cost effective methods such as the disk diffusion test by cefoxitin for MRSA and the D-zone test for MLSBi organisms can easily identify these isolates. Antibiotic resistance profiles from this study can optimize the treatment of multi-drug resistant *S. aureus*.

## Background

*Staphylococcus aureus* is one of the common pathogens isolated in most microbiological laboratories [1]. It is responsible for a wide range of infections including superficial skin infections, food poisoning, osteomyelitis and septicemia [2]. Treatment of infections caused by MRSA is challenging as these organisms are resistant to currently available antibiotics [3]. A lack of newer drugs to keep pace with these superbugs has impelled the precise identification of the organisms and use of the available antibiotics on the basis of antibiotic susceptibility tests. Thus the cautious use of available antibiotics and the addition of newer effective drugs is recommended to treat the multi-drug resistant strains [4].

In Nepal, various laboratories have reported the emergence of multidrug resistant organisms such as extended spectrum beta lactamase producing organisms (ESBL) [5], vancomycin resistant enterococcus (VRE) [6], penicillin resistant *Streptococcus pneumoniae* [7] and MRSA [1, 3, 8].

MRSA is an important group of multidrug resistant organisms responsible for increasing the rate of morbidity and mortality [9]. These organisms are most commonly found in nosocomial infections, however, community-associated MRSA have been detected in recent years in laboratories which are additionally virulent and transmissible [10]. The macrolide group of drugs is generally chosen for oral treatment of these infections which are also the alternative drugs for patients allergic to penicillin. Macrolides such as clindamycin are useful for treating skin and soft tissue infections caused by MRSA [11]. However, emergence of MLSB resistant *S. aureus* has jeopardized the treatment of such cases [12].

The presence of *mec*A gene located on cassette chromosome in *S. aureus* (SCC*mec*) is responsible for methicillin resistance [9]. This gene encodes penicillin binding protein 2A (PBP2A) which has a low affinity for methicillin. The Cefoxitin (30 µg) disk is used to detect MRSA by the disk diffusion method. *S. aureus* that are *mec*A positive should be reported as resistant to oxacillin and other β-lactam antibiotics [13].

There are various methods for detection of MRSA in a microbiology laboratory. Screening of MRSA is commonly performed by molecular and culture methods. In resource limited laboratories, culture is still the efficient method for MRSA identification. The culture method has also been used in Europe as a cost-effective method to detect MRSA [14].

MRSA commonly exhibit resistance to MLSB in addition to many other antibiotics. MLSB resistance is due to methylation of 23S rRNA-binding which is encoded by an *erm* gene. Inducible MLSB resistance (MLSBi) is detected by the D-zone test [12].

To date there have been no studies, particularly in the western region of Nepal to detect MRSA and MLSB. Use of simple and cost effective methods in this study can enhance the identification of these organisms and direct the appropriate treatment. We hypothesized that MRSA and MLSB organisms are prevalent in tertiary level hospitals and are resistant to multiple antibiotics. Thus the specific objective of this study was to determine the prevalence of these organisms and to explore their antibiotic profile.

## Methods

We report a cross-sectional study conducted from September 2015 to February 2016 at Lumbini Medical College and Teaching Hospital (LMCTH), Palpa, Nepal. LMCTH is a tertiary care teaching hospital affiliated with Kathmandu University in Palpa district in the western region of Nepal. This hospital has 600 beds and 20–30 patients get admitted to different departments every day. The hospital serves patients from Palpa and the surrounding districts. The microbiology laboratory in this teaching hospital receives up to 20 samples of culture daily from Out Patient Department (OPD) and wards/In Patient Department (IPD).

### Sample collection

All samples (blood, sputum, urine, pus and body fluids) of patients who attended the hospital for treatment were collected from various departments for culture after the treating clinician requested them. Collected samples were received at the microbiology laboratory for microbiological tests. Sterile containers were used for sample collection using the aseptic technique. The clinicians responsible for the treatment of patients requested all received samples.

### Culture and bacterial identification

Samples were inoculated into McConkey’s agar and blood agar, however, selective media for *S. aureus* was not used. The gram stained smear of the suspected colonies was observed under an oil immersion lens. Gram positive cocci in clusters were subjected to further biochemical tests. *S. aureus* was identified by standard microbiological techniques [2]. Different biochemical tests such as catalase, coagulase and mannitol fermentation tests were performed. Gram positive cocci (GPC) in clusters, which were also catalase positive, were subjected to a slide coagulase test. GPC, which were both catalase and coagulase positive, were considered as *S. aureus.* Those which were negative by the slide coagulase test were further subjected to a tube coagulase test and were considered *S. aureus* if were positive for both the catalase and tube coagulase tests. Finally, all *S. aureus* were confirmed by a mannitol fermentation test.

All *S. aureus* isolated from different samples during the study period were included in the study. However, only first isolate was included in the study if the same patient had other samples (blood, pus, body fluid and sputum) positive for *S. aureus* with same antibiogram. Organisms other than *S. aureus,* including coagulase negative Staphylococcus, were excluded from the study. In addition, various characteristics of patients which included patient’s history, clinical conditions; such as patients with a ventilator, urinary catheter, central line; and patients from different departments such as Intensive Care Unit (ICU) and wards were analyzed for their association with the MRSA infection.

### Antibiotic susceptibility testing of *S. aureus*

Antibiotic susceptibility tests of the *S. aureus* were performed by a modified Kirby–Bauer disk diffusion method according to the guidelines of the Clinical and Laboratory Standards Institute (CLSI 2012) on Mueller–Hinton agar (MHA) [13]. Antibiotic disks (HiMedia Laboratories, Pvt. Limited, India) such as oxacillin (1 μg), cefoxitin (30 μg), penicillin G (10 U), cefazolin (30 μg), cephalexin (30 μg), ceftriaxone (30 μg), ciprofloxacin (5 μg), amoxiclav (20 + 10 μg), tetracycline (30 μg), erythromycin (15 μg), co-trimoxazole (25 μg), gentamicin (10 μg), amikacin (30 μg), clindamycin (2 μg), vancomycin (30 μg), and linezolid (30 µg) were used for antibiotic susceptibility tests.

### Identification of MRSA

Both oxacillin (1 μg) and cefoxitin (30 µg) are used for identification of MRSA. Cefoxitin is considered more accurate than oxacillin. The sensitivity and specificity of the cefoxitin disk to detect MRSA is in concordance with that of *mec*A gene detection by polymerase chain reaction (PCR) [15]. *S. aureus,* which showed a zone of inhibition ≤21 mm with cefoxitin (30 µg) on MHA after overnight incubation at 35 °C, were considered as MRSA [13].

### Identification of MLSB resistant strains

*Staphylococcus aureus* resistant to macrolide, lincosamides and streptogramin B are known as MLSB. Similarly, MLSBi are inducible clindamycin resistant strains which were detected by a disk approximation test. A lawn culture was prepared on MHA with the bacterial suspension equivalent to the turbidity of 0.5 McFarland’s standard. A clindamycin disk (2 μg) was placed 15 mm away from the edge of an erythromycin disk (15 μg) on a MHA plate [13]. After 18–24 h of incubation, organisms that showed flattening of the clindamycin zone of inhibition adjacent to the erythromycin disk (“D” zone) were considered to be MLSBi. A zone size of ≤13 mm around erythromycin and ≤21 mm around clindamycin were considered as resistant. Organisms which were resistant to both antibiotic disks were taken as MLSBc. Organisms were considered as MS (macrolide streptogramin) phenotype when they were resistant to erythromycin and sensitive to clindamycin with a negative D test. Isolates which are sensitive to both erythromycin and clindamycin were reported as erythromycin and clindamycin (ERY, CL) sensitive phenotype [16]. *S. aureus* ATCC 25923 was used as a standard control strain.

### Statistical analysis

Data were analyzed by IBM SPSS statistics 21 software. Frequency and percentage for descriptive and Chi Square test with cross tab for inferential statistics were used.

## Results

A total of 1981 samples (blood 647, pus 188, swab 321, body fluid 354 and urine 471) from patients attending the hospital for treatment were collected and analyzed. From the total of 1981 samples, 133 were confirmed as *S. aureus* and were further tested for MRSA and MLSB. The drug profile of isolates was tested for 11 different antibiotics.

The maximum number of *S. aureus* were found in children <10 years (49.1%) and higher incidence of MRSA infections were found in males (52.4%). However, both failed to elicit a statistically significant difference. High proportion of blood samples contained MRSA (49.2%). A higher proportion (54.2%) of MRSA was derived from ICU. Blood culture is more common among younger patients and children owing to the higher attendance and morbidity (Table 1).

**Table 1:** Variables in relation to MRSA and MSSA (n = 133)

Variables	MRSA (n = 58)	MSSA (n = 75)	p value
	Number (%)	Number (%)	
Age (years)			
<10	26 (49.1)	27 (50.9)	0.452
11–30	16 (36.4)	28 (63.6)	
>30	16 (44.4)	20 (55.6)	
Sex			
Male	33 (52.4)	30 (47.6)	0.057
Female	25 (35.7)	45 (64.3)	
Source of samples			
Ward	27 (48.2)	29 (51.8)	0.307
OPD	14 (33.3)	28 (66.7)	
ER	4 (36.4)	7 (63.6)	
ICU	13 (54.2)	11 (45.8)	
Duration of hospital stay			
No hospital stay	34 (38.6)	54 (61.4)	0.253
<48 h	9 (50.0)	9 (50.0)	
>48 h	15 (55.6)	12 (44.4)	
Type of samples			
Blood	32 (49.2)	33 (50.8)	0.463
Pus	13 (33.3)	26 (66.7)	
Swab	6 (42.9)	8 (57.1)	
Others	7 (46.7)	8 (53.3)	
Patient’s conditions			
Central line			
Opened	21 (56.8)	16 (43.2)	0.079
Not opened	37 (38.5)	59 (61.5)	
On urinary catheter			
Yes	11 (68.8)	5 (31.3)	0.057
No	47 (40.2)	70 (59.8)	
On ventilator			
Yes	4 (57.1)	3 (42.9)	0.699
No	54 (42.9)	72 (57.1)	
ICU stay			
Yes	10 (62.5)	6 (37.5)	0.116
No	48 (41.0)	69 (69.0)	
ET/tracheostomy/NG tube			
Yes	4 (44.4)	5 (55.6)	1
No	54 (43.5)	70 (56.5)	
Surgery before infection			
Yes	8 (61.5)	5 (38.5)	0.24
No	50 (41.7)	70 (58.3)	
Skin lesions			
Yes	11 (44.0)	14 (56.0)	1
No	47 (43.5)	61 (56.5)	
Enteral feeding			
Yes	5 (50.0)	5 (50.0)	0.744
No	51 (42.1)	70 (57.9)	
History of taking antibiotics			
Yes	24 (52.2)	22 (47.8)	0.198
No	34 (39.1)	53 (60.9)	

*Ward* In Patient Department where patients are admitted for treatment, *OPD* Out Patient Department, *ER* emergency, *ICU* Intensive Care unit, *ET tube* endotracheal tube, *NG tube* naso-gastric tube

The cefoxitin disk detected 58 (43.6%) MRSA while oxacillin detected only 43 (32.3%) which was statistically significant (p < 0.001) on post hoc analysis (not shown in the table). MRSA showed a higher degree of resistance to many antibiotics such as erythromycin (62.5%), ciprofloxacin (68.2%), cotrimoxazole (71.4%), gentamicin (73.5%), clindamycin (70.0%) and amikacin (100%) in this study. All the organisms were sensitive to linezolid (Table 2).

**Table 2:** Antibiotic profile of MRSA and MSSA (n = 133)

Antibiotic	MRSA (n = 58)	MSSA (n = 75)	p value
	Number (%)	Number (%)	
Oxacillin			
Sensitive	15 (16.9)	74 (83.1)	<0.001
Resistant	43 (97.7)	1 (2.3)	
Penicillin			
Sensitive	0	10 (100)	0.005
Resistant	58 (47.2)	65 (52.8)	
Ciprofloxacin			
Sensitive	28 (31.5)	61 (68.5)	<0.001
Resistant	30 (68.2)	14 (31.8)	
Cotrimoxazole			
Sensitive	28 (30.8)	63 (69.2)	<0.001
Resistant	30 (71.4)	12 (28.6)	
Gentamicin			
Sensitive	33 (33.3)	66 (66.7)	<0.001
Resistant	25 (73.5)	9 (26.5)	
Amikacin			
Sensitive	48 (39.0)	75 (61.0)	<0.001
Resistant	10 (100)	0	
Tetracycline			
Sensitive	46 (38.7)	73 (61.3)	0.001
Resistant	12 (85.7)	2 (14.3)	
Erythromycin			
Sensitive	18 (26.1)	51 (73.9)	<0.001
Resistant	40 (62.5)	24 (37.5)	
Clindamycin			
Sensitive	37 (37.75)	66 (62.25)	0.001
Resistant	21 (70.0)	9 (30.0)	
Vancomycin			
Sensitive	55 (42.3)	75 (57.7)	0.08
Resistant	3 (100)	0	
Linezolid			
Sensitive	58 (43.6)	75 (56.4)	
Resistant	0	0	

None of the characteristics of the patients (socio-demographic, urinary catheter, ventilator, central line, ICU stay, hospital stay and prior antibiotic use) were found to be associated with isolation of MRSA. MRSA infection was proportionally higher in patients with a central line and urinary catheter, however, the association was not statistically significant.

Out of a total of 64 isolates that showed resistance to erythromycin, 34 were identified as MLSBi by the D-zone test. MLSBi organisms were 100% sensitive to vancomycin and linezolid. Both MLSBi and MLSBc showed higher degree of resistance to multiple antibiotics. Consistently, the same three *S. aureus* were MLSBc as well, showing reduced susceptibility to vancomycin by disc diffusion method (Table 3).

**Table 3:** Antibiotic profile of MLSBi, MLSBc and ERY, CL sensitive phenotype (n = 133)

Antibiotics	MLSB		Sensitive phenotype (69)	p value
	MLSBi (34)	MLSBc (30)		
Oxacillin				
Sensitive	20 (22.5)	13 (14.6)	56 (62.9)	0.001
Resistant	14 (31.8)	17 (38.6)	13 (29.5)	
Penicillin				
Sensitive	1 (10)	2 (20)	7 (70)	0.419
Resistant	33 (26.8)	28 (22.8)	62 (50.4)	
Ciprofloxacin				
Sensitive	22 (24.7)	13 (14.6)	54 (60.7)	0.003
Resistant	12 (27.3)	17 (38.6)	15 (34.1)	
Cotrimoxazole				
Sensitive	20 (22.0)	14 (15.4)	57 (62.6)	0.001
Resistant	14 (33.3)	16 (38.1)	12 (28.6)	
Gentamicin				
Sensitive	26 (26.3)	17 (17.2)	56 (56.6)	0.035
Resistant	8 (23.5)	13 (38.2)	13 (38.2)	
Amikacin				
Sensitive	31 (25.2)	24 (19.5)	68 (55.3)	0.005
Resistant	3 (30.0)	6 (60.0)	1 (10.0)	
Tetracycline				
Sensitive	28 (23.5)	23 (19.3)	68 (57.1)	0.001
Resistant	6 (42.9)	7 (50)	1 (7.1)	
Erythromycin				
Sensitive	0	0	69 (100)	<0.001
Resistant	34 (53.1)	30 (46.9)	0	
Clindamycin				
Sensitive	34 (33)	0	69 (67)	<0.001
Resistant	0	30 (100.0)	0	
Vancomycin				
Sensitive	34 (26.2)	27 (20.8)	69 (53.1)	0.005
Resistant	0	3 (100)	0	
Linezolid				
Sensitive	34 (25.6)	30 (22.6)	69 (51.9)	<0.001
Resistant	0	0	0	

MLSBi, MLSBc and Sensitive phenotype were 25.6, 22.6 and 51.9% of the total respectively. A higher number of MLSB resistant organisms (40/133; 62.5%) were resistant to methicillin (p < 0.001) (Table 4).

**Table 4:** MLSB in relation to MRSA (n = 133)

Variables	MRSA (58)	MSSA (75)	p value
MLSBi	19 (55.9)	15 (44.1)	<0.001
MLSBc	21 (70.0)	9 (30.0)	
Sensitive phenotype	18 (26.1)	51 (73.9)	

## Discussion

This study determined the prevalence of MRSA, MLSBi and MLSBc in LMCTH with their antibiogram. The cefoxitin disc (30 µg) was used to detect MRSA by the disk diffusion method. More than 2 in 5 isolated *S. aureus* were MRSA (43.6%). Findings in our study are consistent with the previous studies conducted in Nepal (Chitwan, 43.1% [1], Kathmandu, 42.4% [3]) and in India (40.2%) [12]. However, varying prevalence of MRSA has been reported from different parts of Nepal such as 26.1% in Dharan [8], 68% in Chitwan [17] and 57.1% in Birgunj [18]. The prevalence of MRSA in this study can alarm the majority of clinical settings, where the beta-lactam group of drugs are extensively used to treat bacterial infections. The development of proportionally high MRSA in these settings might have been due to the wide use of antibiotics available over the counter without specific laboratory tests.

Multidrug resistance patterns were more common in MRSA than MSSA. MRSA was more than 50% resistant to erythromycin, ciprofloxacin and cotrimoxazole. Similarly, a higher degree of resistance to other antibiotics was found in MRSA compared to MSSA. The findings in this study have been consistent with the findings from studies conducted in other parts of Nepal [3, 8].

For MRSA; beta-lactam drugs, beta-lactam/beta-lactamase inhibitor combinations, cephems and carbapenems; should be reported as resistant despite of in vitro susceptibility. However, all cephalosporins except anti-MRSA such as ceftaroline should be reported as resistant irrespective of their zone of inhibition once they are confirmed as MRSA.

On the other hand, methicillin-susceptible *S. aureus* (MSSA) are susceptible to other penicillins, beta-lactam/beta-lactamase inhibitor combinations. Thus routine testing of many beta-lactam drugs can be deduced by testing only penicillin and cefoxitin.

The burden of infections and overuse of antibiotics, (in Nepal all antibiotics are easily available “over the counter”) often without an antibiotic susceptibility test, can easily spread antibiotic resistance across the border, and therefore is a serious threat to the entire world [5, 19, 20]. Immediate strategies against “over the counter antibiotics” through amendment in policy, health education, mandatory antibiotic susceptibility tests before antibiotic prescription, increased funding for antimicrobial resistance through joint collaboration between national, regional and global partners are urgently required.

In this study, socio-demographic and clinical characteristics of patients were analyzed to explore the association with MRSA infection. However, none of these characteristics were found associated with MRSA. Findings in this study are not unique. In recent years, community-associated MRSA infections without any association with the characteristics of patients have been reported [21].

Resistance to the macrolide group of drugs in *S. aureus* has been reported from various parts of the world [11, 12]. In this study, more than 1 in 4 (25.6%) were found as MLSBi among 133 *S. aureus,* which is higher than in the previous study (10.8%) conducted in India [12]. Once the isolate is confirmed as MLSBi, clindamycin is reported as resistant. However, clindamycin could show a good zone of inhibition on MHA when tested independently in the absence of erythromycin. Most of the isolates, which showed MLSBi and MLSBc, were MRSA in this study which is consistent with a study conducted in Libya [22]. Combined resistance patterns (both MLSB and methicillin resistance) is common in *S. aureus* and bears limited treatment options such as oral cotrimoxazole and intravenous vancomycin. Precise identification, timely intervention with the appropriate antibiotic and prevention of transmission can decrease morbidity and mortality of patients in multidrug resistant Staphylococcal infections.

## Limitation

The current study was conducted in one single setting in the western region of Nepal. A multi-setting study within the region and beyond the region could have strengthened the findings. Findings from this study could have been strengthened if MRSA were classified as hospital acquired and community acquired. Nevertheless, the findings in this study can direct the appropriate treatment for a wide variety of infections caused by staphylococcus. The duration of 6 months was chosen arbitrarily to determine the prevalence of MRSA and MLSB resistant organisms in this study. A longer duration of study could have bolstered the findings. Advanced molecular techniques such as PCR could have added to the findings in this study, however, this was beyond the scope of this study.

## Conclusions

This study showed high prevalence of MRSA, MLSBi and MLSBc in a tertiary care hospital in the western region of Nepal. The disk diffusion test by cefoxitin for MRSA and the D-zone test for MLSBi organisms are simple and cost effective methods and can be routinely utilized in resource limited settings to identify these isolates. Antibiotic resistance profiles in this study can direct the optimal treatment for multidrug resistant *S. aureus* infections. Similarly, *S. aureus* showed combined or isolated resistance patterns to methicillin and MLSB. MRSA were resistant to multiple antibiotics except vancomycin and linezolid. This warrants an urgent need of attention to the rational use of vancomycin as a last resort for MRSA. MLSBi detection in this study has demonstrated the limitation of clindamycin use. Further research on these organisms across various settings can explore the level and pattern of resistance over time.

## Abbreviations

MRSA: methicillin resistant *Staphylococcus aureus*
MLSBi: inducible macrolide, lincosamide, streptogramin B resistance
MLCBc: constitutive macrolide, lincosamide, streptogramin B resistance
MS phenotype: macrolide streptogramin phenotype
LMCTH: Lumbini Medical College and Teaching Hospital
CLSI: Clinical and Laboratory Standards Institute
MHA: Mueller–Hinton agar
PCR: polymerase chain reaction
VISA: vancomycin intermediate *Staphylococcus aureus*
VRSA: vancomycin resistant *Staphylococcus aureus*
